# Recent Advances in the Synthesis of Thiophene Derivatives by Cyclization of Functionalized Alkynes

**DOI:** 10.3390/molecules191015687

**Published:** 2014-09-29

**Authors:** Raffaella Mancuso, Bartolo Gabriele

**Affiliations:** Dipartimento di Chimica e Tecnologie Chimiche, Università della Calabria, Via P. Bucci, 12/C, Arcavacata di Rende (CS) 87036, Italy

**Keywords:** alkynes, catalysis, cyclization, heterocyclization, thiophenes

## Abstract

This review is intended to highlight some recent and particularly interesting examples of the synthesis of thiophene derivatives by heterocyclization of readily available *S*-containing alkyne substrates.

## 1. Introduction

Substituted thiophenes are among the most important aromatic heterocyclic derivatives. Many molecules incorporating the thiophene nucleus have, in fact, shown important pharmacological activities [1,2,3,4]. Moreover, thiophene derivatives find large application in material science [5,6,7,8,9,10,11,12,13,14,15] and in coordination chemistry [16,17], and as intermediate in organic synthesis [18,19].

The classical approaches to substituted thiophenes are mainly based on condensation-like reactions or on subsequent functionalization of the thiophene ring [20,21,22,23,24,25,26,27,28,29,30]. However, during the last years, innovative approaches to the regioselective synthesis of substituted thiophenes starting from acyclic precursors have been developed, mainly based on heterocyclization of functionalized alkynes [31].

In this review, we will highlight some recently developed efficient and selective syntheses of thiophene derivatives by cyclization of readily available *S*-containing alkyne substrates, which have allowed a significant step forward toward a direct and atom-economical entry to this very important class of aromatic heterocycles. As a matter of fact, these processes may allow the construction of the thiophene ring with the desired substitution pattern in a regiospecific manner and in only one step, usually with high atom economy (particularly in the case of cycloisomerization reactions), and starting from readily available starting materials (as the acetylenic *S*-contaning precursors can be easily prepared in a few step from commercially available compounds through simple synthetic steps).

As will be seen, many of these cyclization reactions leading to thiophenes have been performed under mild conditions (even at rt, in particular with iodocyclizations) in classical organic solvents, either dipolar aprotic (such as *N*,*N*-dimethylacetamide (DMA), dimethylsulfoxide (DMSO), or MeCN), apolar or slightly polar (such as toluene, THF, or CH_2_Cl_2_), or protic ones (such as MeOH). However, particularly during the last years, the possibility to carry out these processes in unconventional solvents, such as ionic liquids (ILs) has been successfully verified. This has allowed the easy and convenient recycling of the reaction medium and/or of the catalyst (in the case of metal-catalyzed heterocyclizations).

We have structured the review into different Sections. Section 2 will deal with metal-catalyzed or base-promoted heterocyclizations, while in Section 3 iodocyclization reactions will be discussed. Carbocyclization of *S*-containing alkyne substrates is the topic of Section 4. In Section 5, some miscellaneous methods that cannot be classified into the previous categories are treated. We would like to point out here that most of the mechanisms shown in this review are based on mechanistic pathways proposed by the authors, on the basis of the existing knowledge and, in some cases, of some additional experimental evidences (product stereochemistry, reactivity pattern of the substrates, and so on). Only in a few cases these hypotheses have been corroborated by computation calculations (one example is the iodocyclization of 1-mercapto-3-yn-2-ols **23** in ionic liquids, Section 3, while, to the best of our knowledge, no kinetic studies have been reported so far. Another aspect worth mentioning concerns the reaction conditions reported in the review: they refer to the optimized conditions, usually established after a careful study on the influence of the reaction parameters (such as the catalyst loading, reagents molar ratios, solvent, temperature and so on) on substrate reactivity and product yield.

## 2. Synthesis of Thiophene Derivatives by Metal-Catalyzed or Base-Promoted Heterocyclization of *S*-Containing Alkyne Substrates

**Scheme 1:**
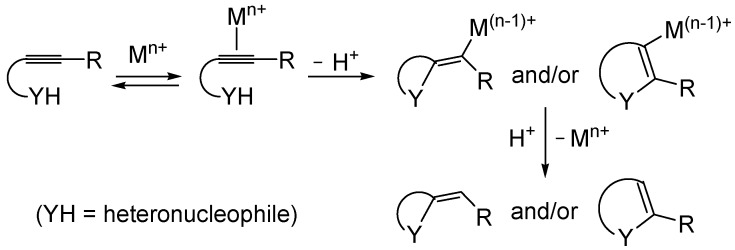
Metal-catalyzed heterocyclization of functionalized alkynes bearing a suitably placed nucleophilic group leading to heterocycles through activation of the triple bond by the metal species followed by intramolecular nucleophilic attack by the heteronucleophile and protonolysis.

Metal-catalyzed heterocyclization of functionalized alkynes bearing a suitably placed heteronucleophilic group is a powerful methodology for the regioselective and atom-economical synthesis of substituted heterocycles starting from readily available acyclic substrates (Scheme 1, Y = heteroatom) [31,32,33,34,35,36,37,38,39,40,41,42,43,44,45,46,47,48,49]. The generally accepted mechanism for this important transformation involves the electrophilic activation of the triple bond by coordination to the metal center, followed by either *exo* or *endo* cyclization (ensuing from intramolecular nucleophilic attack by the –YH group to the coordinated triple bond) and protonolysis (Scheme 1).

Compared to the considerable number of examples reported in the literature of the synthesis of *O*- or *N*-heterocycles (Scheme 1, Y = O or N), there are still relatively few examples of metal-catalyzed heterocyclizations of *S*-containing alkyne substrates leading to sulfur heterocycles (Scheme 1, Y = S).

This is probably connected with the “poisoning” effect exerted by the sulfur atom on the metal catalyst, owing to its strong coordinating and adsorptive properties [50,51]. Nevertheless, progress in organometallic catalysis has recently permitted to develop several important processes involving the metal-catalyzed carbon-sulfur bond formation [52,53,54,55]. Although most of these processes concern the formation of sulfurated acyclic molecules, during the last years several important *S*-cyclization reactions, involving the formation of the C-S bond and leading to S-heterocycles, have been developed.

The first example of the formation of thiophenes by a metal-catalyzed cycloisomerization approach of alkynylthiol derivatives was reported in 2000 by our research group [56]. It concerned the reaction of (*Z*)-2-en-4-yne-1-thiols **1** (readily obtainable from the corresponding (*Z*)-2-en-4-yn-1-ols [57]) in *N*,*N*-dimethylacetamide (DMA) as the solvent or under solventless conditions at 25–100 °C, carried out in the presence of a particularly simple catalytic system, consisting of PdI_2_ (1 mol %) in conjunction with KI (2 mol %) (Table 1). The use of KI was necessary in order to make PdI_2_ soluble and to stabilize the formation of the catalytically active species PdI_4_^2−^. With low-boiling substrates, solventless conditions were used to facilitate product recovery. In other cases, several polar solvent were tested (a polar solvent was necessary to ensure the dissolution of the ionic catalyst), and, between them, DMA gave the best results in terms of substrate reactivity and product yield. Substrates bearing a terminal as well as an internal triple bond could be employed, while alkyl as well aryl substitution was tolerated on the double bond and at C-1 (Table 1) [56]. One indubitable advantage of this new protocol consisted in the practically neutral conditions employed for realizing the thiocyclization, as compared with the strongly basic conditions previously used (*t*-BuOK in *t*-BuOH in the presence of 18-crown-6), which were not compatible with base-sensitive substrates such as those bearing a terminal triple bond [58].

**Scheme 2:**
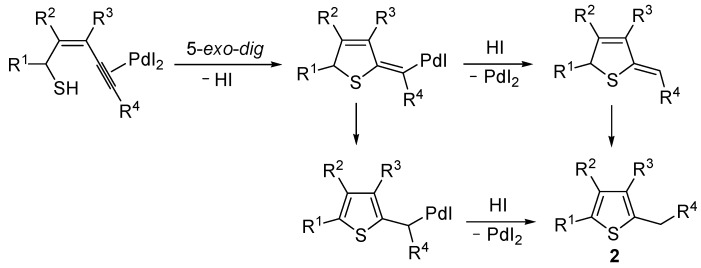
Proposed mechanistic pathways for the PdI_2_-catalyzed cycloisomerization of (*Z*)-2-en-4-yne-1-thiols **1** leading to thiophenes **2** [56].

Mechanistically, the reaction is believed to proceed through *anti* 5-*exo*-*dig* intramolecular nucleophilic attack by the thiolic group to the triple bond coordinated to Pd(II), with formal elimination of HI, followed by protonolysis and aromatization or vice versa (Scheme 2; anionic iodide ligand are omitted for clarity). This mechanistic hypothesis was in agreement with the experimental observation that substrates bearing a terminal triple bond were more reactive with respect to those bearing an internal triple bond. With an internal triple bond, in fact, Pd(II) coordination is less favored for steric reasons. A nucleophilic attack by the –SH group on the triple bond, with Pd(II) being coordinated from the opposite site (*anti* attack), was also in agreement with the significantly higher reactivity observed with substrates unsubstituted at C-3 with respect to enynethiols substituted at C-3 (Table 1, compare Entries 5 and 6). This is clearly related to the fact that an *anti*-coordination of the triple bond to Pd(II) may be less efficient, for steric reasons, in the presence of a substituent at C-3 [56].

**Table 1:** PdI_2_/KI-catalyzed cycloisomerization of (*Z*)-2-en-4-yne-1-thiols **1** to substituted thiophenes **2** *^a^* [56]. 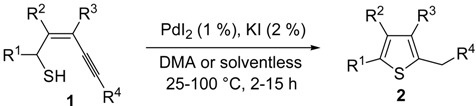

Entry	1	T (°C)	Solvent	t (h)	2	Yield of 2 *^b^* (%)
1 *^c^*	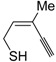	100	none	2	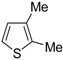	36
2	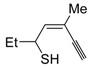	100	none	1	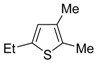	71
3	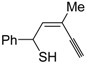	100	DMA	1.5	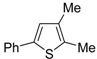	58
4	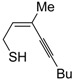	100	DMA	15	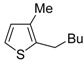	44
5	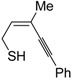	100	DMA	8	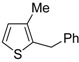	56
6	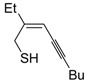	25	DMA	1	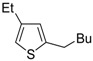	89

*^a^*: Unless otherwise noted, all cycloisomerization reactions were carried out under nitrogen using **1**:KI:PdI_2_ molar ratio of 100:2:1. For the reactions carried out in DMA, substrate concentration was 2 mmol of **1** per mL of DMA; *^b^*: Isolated yield based on starting **1**; *^c^*: The reaction was carried out with 2 mol % of PdI_2_.

**Scheme 3:**
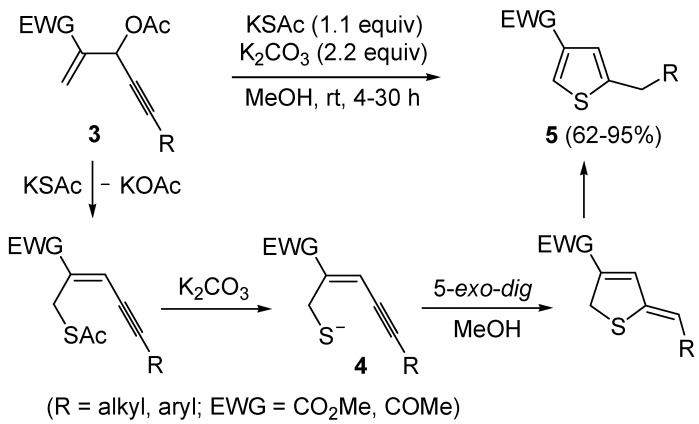
Synthesis of 2,4-disubstitued thiophenes **5** from 4-en-1-yn-3-yl acetates **3** by sequential allylic nucleophilic substitution with KSAc followed by base-promoted deacylation, to give (*Z*)-2-en-4-yne-1-thiolate derivatives **4**, and base-promoted thiocyclization [59].

More recently, a strictly related method has been published, regarding the metal-free cyclization of 4-en-1-yn-3-yl acetates **3** to give 2,4-disubstituted thiophenes **5** through the intermediate formation of (*Z*)-2-en-4-yne-1-thiolate derivatives **4**, formed* in situ* by allylic nucleophilic substitution with KSAc followed by base-promoted deacylation (Scheme 3) [59]. Intermediates **4** were then converted into thiophenes **5** by 5-*exo*-*dig* cyclization and aromatization. The reaction has been applied to the synthesis of several 2,4-disubstitued thiophenes, but presented limitations due to the strong basic conditions employed (for example, the reaction could not be applied to substrates bearing a terminal triple bond) and to the need for the presence of an electron-withdrawing group (EWG) at the C-4 of the starting material (Scheme 3) [59].

**Scheme 4:**
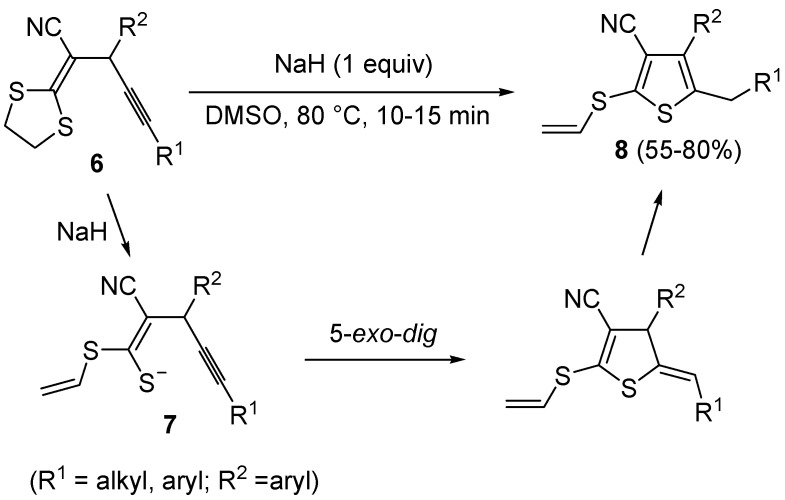
Synthesis of 3-cyano-2-(vinylthio)thiophenes **8** from 2-(1,3-dithiolan-2-ylidene)-4-ynenitriles **6** by NaH-induced ring opening, to give (*Z*)-1-en-4-yne-1-thiolates **7**, followed by 5-*exo*-*dig* cyclization and aromatization [60].

**Scheme 5:**
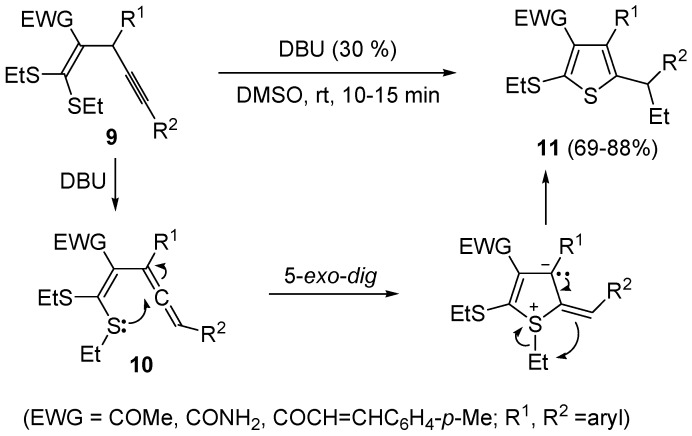
Synthesis of thiophenes **11** from 1,1-*bis*(ethylthio)-1-en-4-ynes **9** by DBU-induced isomerization to *gem*-dialkylthiovinylallenes **10** followed by 5-*exo*-*dig* *S*-cyclization and 1,3-migration of the ethyl group [61].

In a similar way, 3-cyano-2-(vinylthio)thiophenes **8** were obtained from 2-(1,3-dithiolan-2-ylidene)-4-ynenitriles **6** by NaH-induced ring opening of the 1,3-dithiolan-2-ylidene group (ensuing from deprotonation of the methylene moiety bonded to sulfur), leading to the corresponding (*Z*)-1-en-4-yne-1-thiolates **7**, followed by 5-*exo*-*dig* cyclization and aromatization (Scheme 4) [60]. Interestingly, the similar substrates 1,1-*bis*(ethylthio)-1-en-4-ynes **9**, bearing an electron withdrawing group, such as the carbonyl, at the 2 position, reacted in a rather different way when treated with a base such as DBU. In this case, in fact, the intermediate formation of *gem*-dialkylthiovinylallenes **10** took place, followed by 5-*exo*-*dig* *S*-cyclization and 1,3-migration of the ethyl group from sulfur to the benzylic carbon, eventually leading to substituted thiophenes **11** in good to high yields (Scheme 5) [61].

**Scheme 6:**
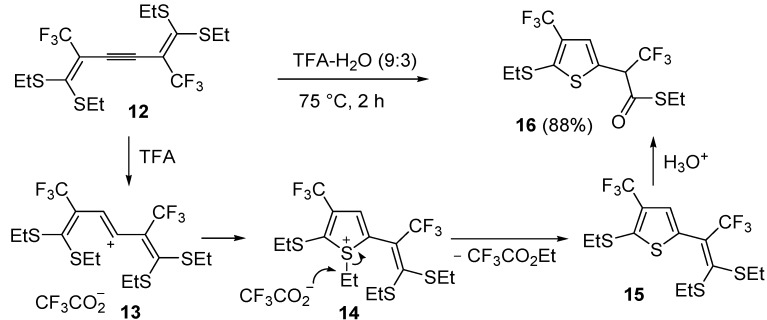
Synthesis of *S*-ethyl 2-(5-(ethylthio)-4-(trifluoromethyl)thiophen-2-yl)-3,3,3-trifluoropropanethioate (**16**) from 1,1,6,6-*tetrakis*(ethylthio)-2,5-*bis*(trifluoromethyl)hexa-1,5-dien-3-yne (**12**) through the intermediate formation of 5-(3,3-*bis*(ethylthio)-1,1,1-trifluoroprop-2-en-2-yl)-2-(ethylthio)-3-(trifluoromethyl)thiophene (**15**) [62].

An interesting approach to multifunctionalized thiophene **16** from 1,1,6,6-*tetrakis*(ethylthio)-2,5-*bis*(trifluoromethyl)hexa-1,5-dien-3-yne (**12**) has been reported, based on treatment of **12** with a mixture of trifluoroacetic acid and water (TFA-H_2_O 9:3) at 75 °C for 2 h (Scheme 6) [62]. 

**Scheme 7:**
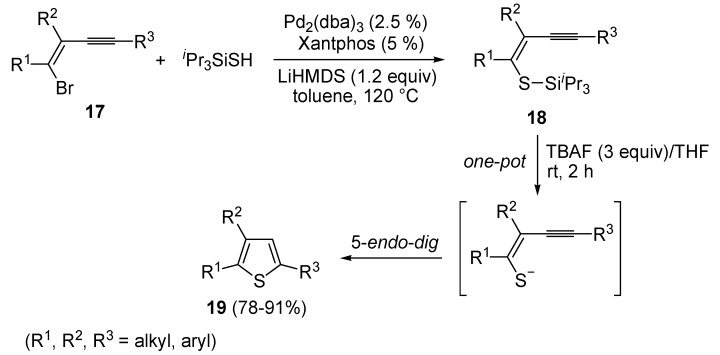
Synthesis of thiophenes **19** from (*Z*)-1-bromo-1-en-3-ynes **17** by Pd-catalyzed coupling with triisopropylsilanethiol, to give (*Z*)-(1-en-3-ynylthio)triisopropylsilanes **18**, followed by one-pot 5-*endo*-*dig* cyclization, induced by desilylation with tetrabutylammonium fluoride (TBAF) [63].

Formation of *S*-ethyl 2-(5-(ethylthio)-4-(trifluoromethyl)thiophen-2-yl)-3,3,3-trifluoropropane-thioate (**16**) is explained to occur by triple bond protonation to give stabilized carbocation **13** followed by sulfur attack to the carbocation and nucleophilic attack of the trifluoroacetate anion to the resulting sufonium cation **14**. This leads to the formation of 5-(3,3-*bis*(ethylthio)-1,1,1-trifluoroprop-2-en-2-yl)-2-(ethylthio)-3-(trifluoromethyl)thiophene intermediate (**15**), which has been isolated under appropriate conditions, and which upon hydrolysis leads to the final product **16** (Scheme 6) [62]. A one-pot C-S coupling/heterocyclization approach to substituted thiophenes **19** has been recently reported [63]. It involves the Pd-catalyzed reaction of (*Z*)-1-bromo-1-en-3-ynes **17** with triisopropylsilanethiol (1.2 equiv), carried out in the presence of Xantphos as ligand and lithium hexamethyldisilazane (LiHMDS) as the base, to give (*Z*)-(1-en-3-ynylthio)triisopropylsilanes **18**, followed by 5-*endo*-*dig* cyclization, induced by desilylation with tetrabutylammonium fluoride (TBAF) (Scheme 7) [63]. 

**Scheme 8:**
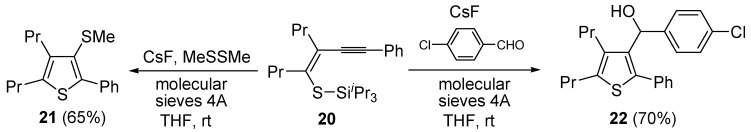
Synthesis of 3-(methylthio)-2-phenyl-4,5-dipropylthiophene (**21**) and (4-chlorophenyl)(2-phenyl-4,5-dipropylthiophen-3-yl)methanol (**22**) from (*Z*)-triisopropyl(5-(2-phenylethynyl)oct-4-en-4-ylthio)silane (**20**) by CsF-induced 5-*exo*-*dig* *S*-cyclization in the presence of a suitable electrophile (dimethyldisulfide or *p*-chlorobenzaldehyde respectively) [63].

Further functionalization of the final product, to give thiophenes **21** and **22**, could be introduced by reacting purified (*Z*)-triisopropyl-(5-(2-phenylethynyl)oct-4-en-4-ylthio)silane (**20**) with CsF in the presence of a suitable electrophile, such as dimethyldisulfide or *p*-chlorobenzaldehyde, and molecular sieves 4A (Scheme 8) [63].

**Scheme 9:**
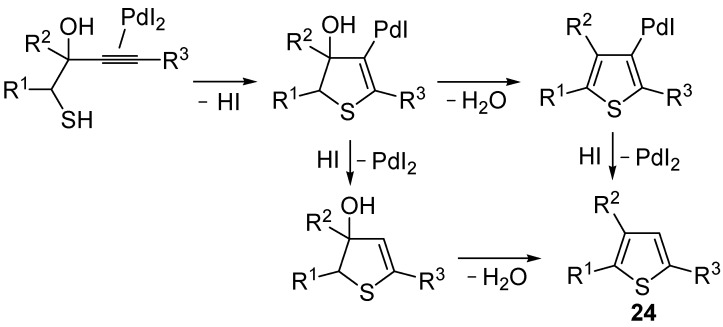
Proposed mechanistic pathways for the PdI_2_-catalyzed heterocyclodehydration of 1-mercapto-3-yn-2-ols **23** leading to thiophenes **24** [64].

A 5-*endo*-*dig* *S*-cyclization was also involved in the synthesis of substituted thiophenes **24** by Pd-catalyzed heterocyclodehydration of readily available 1-mercapto-3-yn-2-ols **23**, recently reported by our research group [64]. The cyclization reaction is catalyzed by PdI_2_ in conjunction with an excess (10:1 molar ratio) of KI, and takes place either in MeOH at 50–100 °C (Table 2) or in an ionic liquid, such as BmimBF_4_, at 80 °C. These are optimized conditions, after a careful study on the influence of the reaction parameters (such as the KI:PdI_2_ molar ratio, the catalyst loading, and so on) on substrate reactivity and product yield. In the case of the reactions carried out in BmimBF_4_, the catalyst-solvent system could be recycled several times without appreciable loss of activity (Table 3) [64]. This protocol generalized the previous finding by Aponick and coworkers, who reported the Au/Ag-catalyzed transformation of 1-mercapto-4-phenylbut-3-yn-2-ol into 2-phenylthiophene, carried out using 5 mol % of Au[P(*t*-Bu)_2_(*o*-biphenyl)]Cl and 5 mol % of AgOTf in THF at 40 °C in the presence of molecular sieves 4A [65]. The heterocyclodehydration process takes place by 5-*endo*-*dig* intramolecular nucleophilic attack of the thiol group to the triple bond coordinated to the metal center, with elimination of HI, followed by dehydration and protonolysis or *vice versa* (Scheme 9; anionic iodide ligands are omitted for clarity) [64].

**Table 2:** PdI_2_/KI-catalyzed heterocyclodehydration of 1-mercapto-3-yn-2-ols **23** to substituted thiophenes **24** *^a^* [64]. 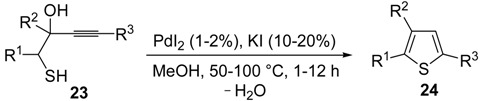

Entry	23	PdI_2_ (mol %)	T (°C)	t (h)	24	Yield of 24 (%) *^b^*
1	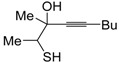	1	50	3	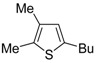	88
2	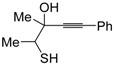	1	80	1	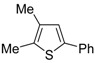	89
3	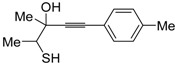	2	50	12	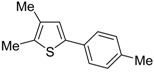	75
4	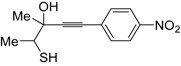	1	50	1	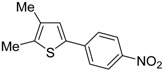	80
5	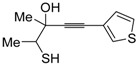	1	50	24	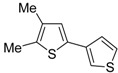	78
6	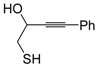	2	100	6	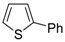	50
7	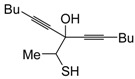	1	50	8	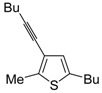	85
8	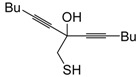	1	50	8	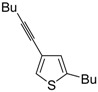	52 *^c^*
9	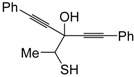	2	80	3	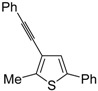	85

*^a^*: All cycloisomerization reactions were carried out in MeOH as the solvent (0.5 mmol of starting thiol **23** per mL of MeOH) in the presence of PdI_2_ and KI (KI:PdI_2_ molar ratio of 10). Conversion of substrate was quantitative; *^b^*: Isolated yield based on starting **23**; *^c^*: Substrate conversion was 74%.

**Table 3:** Recyclable catalytic synthesis of substituted thiophenes **24** by PdI_2_/KI-catalyzed heterocyclodehydration of 1-mercapto-3-yn-2-ols **23** in BmimBF_4_*^a^* [64]. 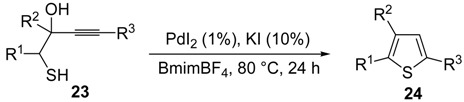

Entry	23	24	Yield of 24 (%) *^b^*						
			Run 1	Run 2	Run 3	Run 4	Run 5	Run 6	Run 7 *^c^*
1	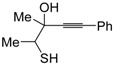	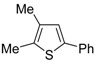	81	83	80	79	79	78	79
2	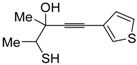	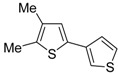	71	72	71	70	70	71	69
3	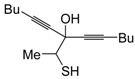	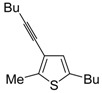	78	77	77	76	76	77	76
4	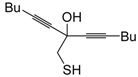	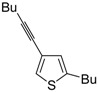	67	66	67	66	66	65	65

*^a^*: All cycloisomerization reactions were carried out at 80 °C for 24 h in BmimBF_4_ as the solvent (0.2 mmol of starting thiol **23** per mL of BmimBF_4_) in the presence of PdI_2_ (1 mol %) and KI (KI:PdI_2_ molar ratio of 10). Conversion of substrate was quantitative; *^b^*: Isolated yield based on starting **23**; *^c^*: Run 1 corresponds to the 1st experiment, the next runs to recycles.

**Scheme 10:**
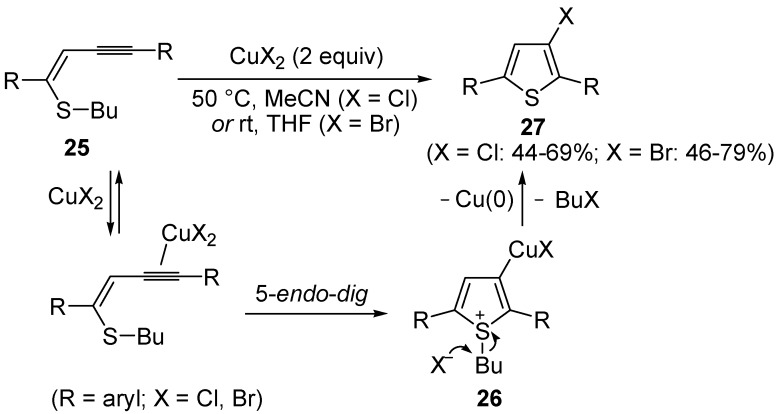
Synthesis of thiophenes **27** from (*Z*)-1-en-3-ynyl(butyl)sulfanes **25** by CuX_2_-promoted 5-*endo*-*dig* *S*-cyclization, to give the sulfonium salt **26**, followed by elimination of BuX and Cu(0) [66].

Copper-promoted or –catalyzed cyclization of *S*-containing alkyne derivatives to give thiophenes has also been reported. Thus, (*Z*)-1-en-3-ynyl(butyl)sulfanes **25** were converted into the corresponding substituted 3-halothiophenes **27** (X = Cl or Br) when treated with 2 equiv of CuX_2_ in MeCN (X = Cl) or THF (X = Br) as the solvent (Scheme 10) [66]. The process is believed to proceed through CuX_2_-promoted 5-*endo*-*dig* *S*-cyclization, to give the sulfonium salt **26**, followed by reductive elimination with simultaneous nucleophilic attack by the X^−^ anion to the butyl group bonded to the sulfur atom of **26** (Scheme 10) [66].

**Scheme 11:**
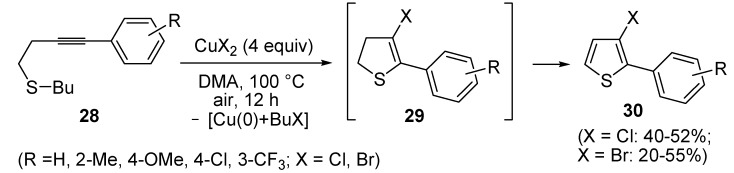
Synthesis of 2-aryl-3-halothiophenes **30** from but-3-ynyl(butyl)sulfanes **28** by CuX_2_-mediated 5-*endo*-*dig* *S*-cyclization, elimination of BuX and Cu(0), and* in situ* oxidation of dihydrothiophene intermediates **29** [67].

In a strictly related process, 2-aryl-3-halothiophenes **30** were obtained in moderate yields from but-3-ynyl(butyl)sulfanes **28**, when working in the presence of 4 equiv of CuX_2_ (X = Cl, Br) in DMA under air at 100 °C for 12 h, through the intermediate formation of dihydrothiophenes **29** (Scheme 11) [67].

The Cu(I)-catalyzed tandem addition of terminal alkynes to 1-phenylsulfonylalkylidenethiiranes **31**/cycloisomerization has allowed a convenient synthesis of functionalized thiophenes **33** (Table 4) [68].

**Table 4:** CuCl/DBU-catalyzed tandem addition/cycloisomerization of 1-phenylsulfonylalkylidenethiiranes **31** with terminal alkynes leading to thiophenes **33** *^a^* [68]. 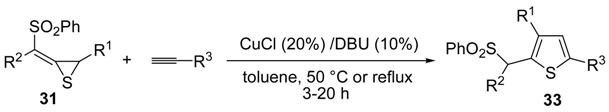

Entry	31	1-Alkyne	t (h)	33	Yield of 33 (%) *^b^*
1	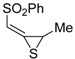	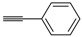	10	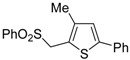	91
2	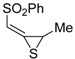	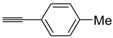	10	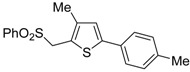	86
3	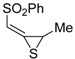	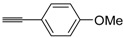	8	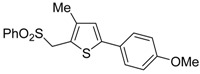	68
4	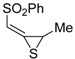	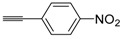	10	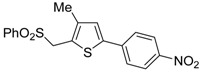	65
5	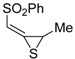	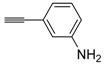	10	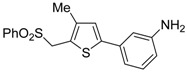	67
6	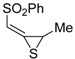	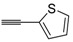	12	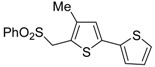	68
7	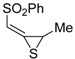	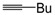	5	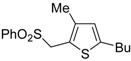	91
8 *^c^*	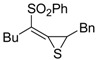	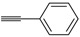	14	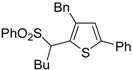	76
9 *^c^*	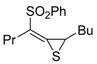	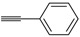	16	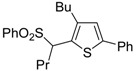	72
10 *^c^*	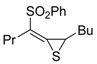	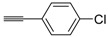	20	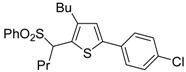	68

*^a^*: All reactions were carried out in toluene at 50 °C in the presence of CuCl (20 mol %) and DBU (10 mol %), with a **31**: alkyne molar ratio of 1:1.5; *^b^*: Isolated yield based on starting **31**; *^c^*: The reaction was carried out under reflux.

**Scheme 12:**
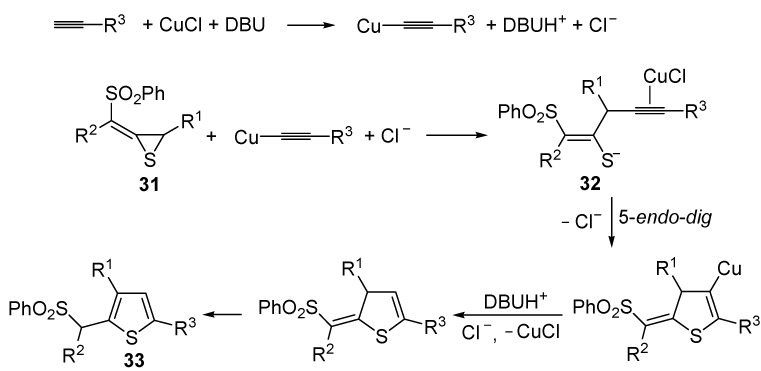
Formation of thiophenes **33** by CuCl/DBU-catalyzed tandem addition/cycloisomerization of 1-phenylsulfonylalkylidenethiiranes **31** with terminal alkynes through the intermediate formation of (*Z*)-1-phenylsulfonyl-1-en-4-yne-2-thiolate **32** [68].

Reactions were carried out in toluene at 50 °C or under reflux with a molar ratio of **31**: alkyne:CuCl of 1:1.5:0.2, in the presence of DBU (10 mol % with respect to **31**). The reaction is believed to proceed through base-promoted formation of an alkynylcopper intermediate, whose regiospecific attack to the C-2 of the thiirane ring affords (*Z*)-1-phenylsulfonyl-1-en-4-yne-2-thiolate intermediate **32**. 5-*Endo*-*dig* cyclization of the latter, ensuing from intramolecular nucleophilic attack of the thiolate group to the triple bond coordinated to CuCl, followed by protonolysis and isomerization, eventually leads to the final thiophene derivative **33** (Scheme 12) [68].

**Scheme 13:**
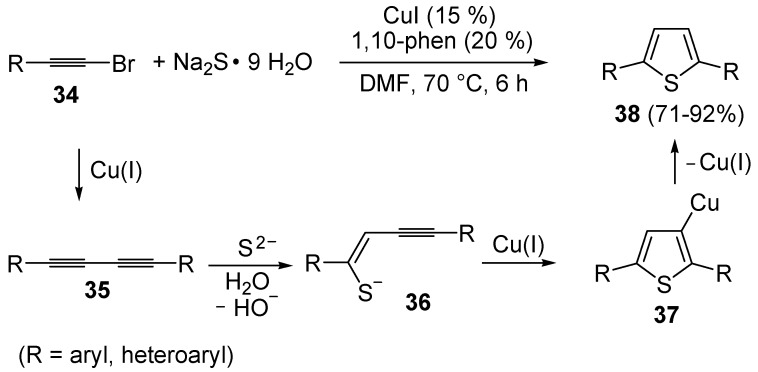
Synthesis of thiophenes **38** from 1-bromoalkynes **34** and Na_2_S by CuI-induced 5-*endo*-*dig* *S*-cyclization of enynethiolate intermediate **36**, formed by sulfide addition to 1,3-diynes **35**, deriving in their turn from CuI-catalyzed homocoupling of **34** [69].

An interesting approach to 2,5-disubstituted thiophenes **38**, starting from 1-bromoalkynes **34** and Na_2_S (5 equiv) in the presence of CuI (15 mol %) and 1,10-phenanthroline (20 mol %), in DMF at 70 °C, has been recently developed (Scheme 13) [69]. The proposed mechanism starts with the Cu(I)-catalyzed formation of 1,3-diynes **35**, followed by sulfide attack to the triple bond to give enynethiolate intermediate **36**. Cu-promoted 5-*endo*-*dig* cyclization of the latter, ensuing from intramolecular attack by the sulfur atom to the triple bond coordinated to CuI, leads to 3-thienylcopper complex **37**, from which the final product is formed by protonolysis (Scheme 13) [69].

**Scheme 14:**
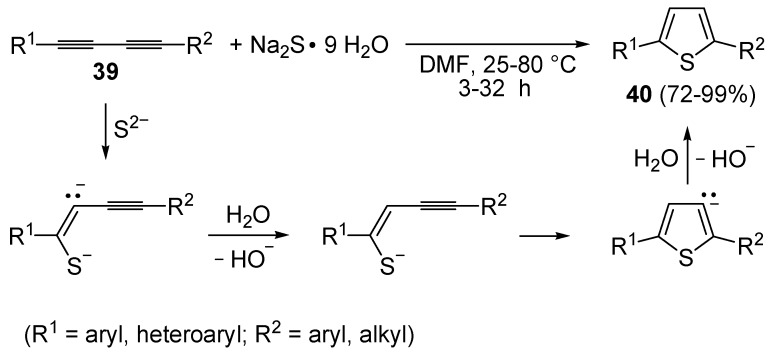
Metal-free synthesis of thiophenes **40** from 1,3-diynes **39** and Na_2_S [70].

The direct, metal-free conversion of 1,3-diynes **39** to thiophenes **40**, by reaction with a 3-fold excess of NaSH or Na_2_S•9H_2_O in DMF at 25–80 °C for 1–48 h, has also been reported, as exemplified in Scheme 14 [70].

## 3. Synthesis of Thiophene Derivatives by Iodocyclization of *S*-Containing Alkyne Substrates

**Scheme 15:**
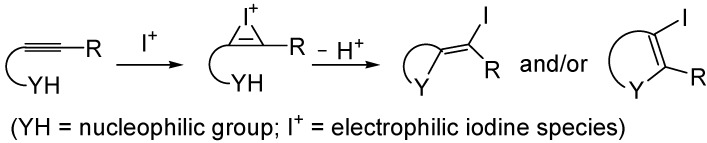
Iodocyclization of alkynes bearing a suitably placed nucleophilic group leading to iodinated carbo- or heterocycles [71,72,73,74,75,76,77,78,79].

The iodocyclization of suitably functionalized alkynes is a very important synthetic tool for the direct preparation of iodine-containing carbo- and heterocycles starting from readily available starting materials [71,72,73,74,75,76,77,78,79]. The utility of the method is further demonstrated by the possibility to elaborate the final products through various cross-coupling reactions (such as Heck, Suzuki-Miyaura, and Sonogashira reactions). The process is usually carried out under mild conditions, and takes place trough intramolecular nucleophilic attack of the nucleophilic group of the substrate to the iodonium ion formed by the reaction between the triple bond and the electrophilic iodine species (indicated with I^+^); both *exo* and *endo* cyclization modes are possible, as shown in Scheme 15. The process is usually carried out in the presence of a base, to buffer the acid generated during the process.

**Scheme 16:**
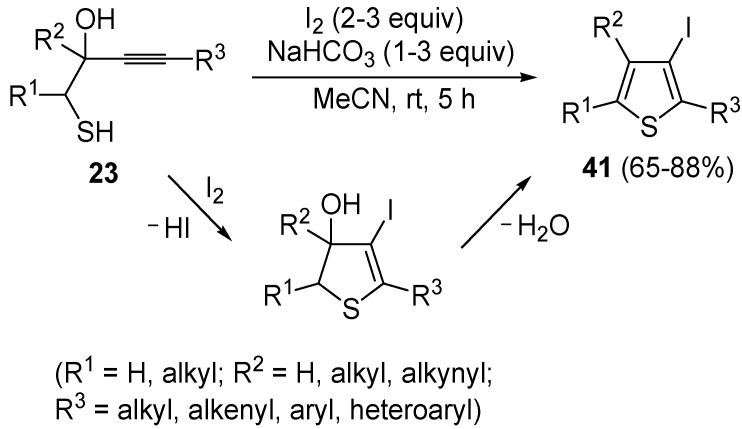
Synthesis of 3-iodothiophenes **41** by 5-*endo*-*dig* iodocyclization/dehydration of 1-mercapto-3-yn-2-ols **23** [86].

Recently, several novel approaches to iodinated thiophenes have been reported, starting from readily available sulfur-containing alkynes. As an extension of the previously reported syntheses of 3-iodofuran and 3-iodopyrrole derivatives by the iodocyclization of 3-yne-1,2 diols [80,81,82,83,84] and *N*-protected 1-amino-3-yn-1-ols [80,85], respectively, our research group has reported a particularly facile and convenient synthesis of 3-iodothiopenes **41** by dehydrative iodocyclization of 1-mercapto-3-yn-2-ols **23**, according to Scheme 16 [86]. Reactions were carried out in MeCN at room temperature for 5 h, using molecular iodine as the electrophilic iodine species (2–3 equiv) and NaHCO_3_ as the base (1–3 equiv). Iodine-induced 5-*endo*-*dig* cyclization was followed by dehydration with aromatization to give **41** in fair to high yields (65%–88%, Scheme 16) [86].

Interestingly, the process also took place in an ionic liquid bearing a basic anionic moiety, such as 1-ethyl-3-methylimidazolium ethylsulfate (EmimEtSO_4_), as the solvent, in the absence of external bases [87]. As reported in Table 5, the reaction medium could be recycled several times without significantly affecting the reaction outcome. Theoretical calculations have confirmed the role of the ethylsulfate anion in the deprotonation of the thiolic group of the substrate [87].

**Scheme 17:**
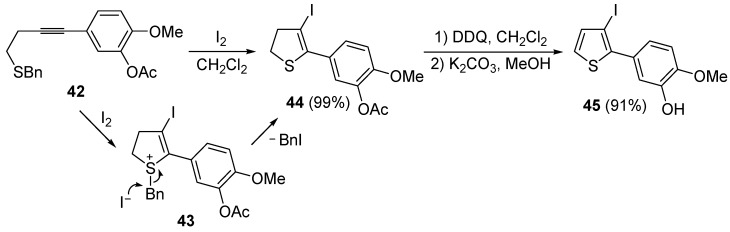
Synthesis of 5-(3-iodothiophen-2-yl)-2-methoxyphenol **45** by 5-*endo*-*dig* iodocyclization of 5-(4-(benzylthio)but-1-ynyl)-2-methoxyphenyl acetate **42** to give 5-(3-iodo-4,5-dihydrothiophen-2-yl)-2-methoxyphenyl acetate **44** (through the intermediate formation of sulfonium ion salt **43**) followed by oxidation and deacylation [88].

A thioether or thioester group can also act as intramolecular nucleophile in an iodocyclization process, eventually leading to a thiophene derivative. Thus, 5-(4-(benzylthio)but-1-ynyl)-2-methoxyphenyl acetate **42** was easily transformed into 5-(3-iodothiophen-2-yl)-2-methoxyphenol **45** in almost quantitative yield by a three-step procedure, involving iodocyclization (carried out with I_2_ in CH_2_Cl_2_ at room temperature), to give dihydrothiophene **44**, followed by oxidation with DDQ and deacylation with K_2_CO_3_ in MeOH (Scheme 17) [88]. 

**Table 5:** Recyclable and base-free synthesis of 3-iodothiophenes **41** by iodoheterocyclization of 1-mercapto-3-yn-2-ols **23** in EmimEtSO_4_ *^a^* [87]. 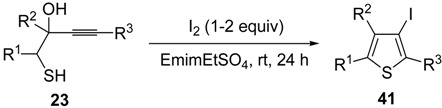

Entry	23	41	Yield of 41 (%) *^b^*						
			Run 1	Run 2	Run 3	Run 4	Run 5	Run 6	Run 7 *^c^*
1	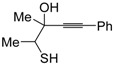	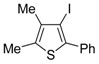	70	68	70	71	72	70	72
2	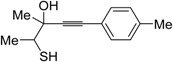	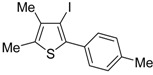	65	67	68	62	65	65	60
3	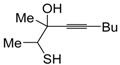	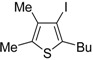	77	68	67	69	68	70	71
4 *^d^*	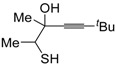	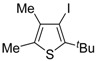	77	73	70	65	61	60	60
5 *^e^*	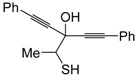	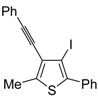	81	73	76	75	72	73	74

*^a^*: Unless otherwise noted, all iodocyclization reactions were carried out at rt for 24 h in EmimEtSO_4_ as the solvent (0.25 mmol of starting thiol **23** per mL of EmimEtSO_4_) in the presence of 1 equiv of I_2_. Conversion of substrate was quantitative; *^b^*: Isolated yield based on starting **23**; *^c^*: Run 1 corresponds to the 1st experiment, the next runs to recycles; *^d^*: The reaction was carried out with a I_2_:substrate molar ratio of 2; *^e^*: The reaction was carried out with a I_2_:substrate molar ratio of 1.5.

As concerns the mechanism leading to **44**, as shown in Scheme 17, the initial iodocyclization is followed by nucleophilic attack by the iodide anion on the benzyl group of the sulfonium intermediate **43**. In a similar way, (*Z*)-1-en-3-ynyl(butyl)sulfanes **46** were smoothly converted into 3-iodothiophenes **47** when treated with 1.1 equiv of I_2_ in CH_2_Cl_2_ or 1,2-dichloroethane (DCE) at rt or 70 °C for 5 min–2 h, as shown in Table 6 [89,90].

**Table 6:** Synthesis of 3-iodothiophenes **47** by iodocyclization of (*Z*)-1-en-3-ynyl(butyl)sulfanes **46** *^a^* [89,90]. 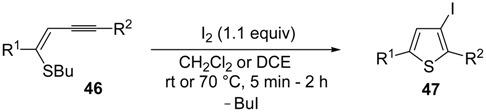

Entry	46	Solvent	t	T	47	Yield of 47 (%) *^b^*
1	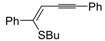	CH_2_Cl_2_	5 min	rt	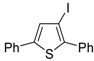	82
2	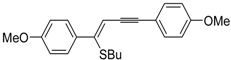	CH_2_Cl_2_	5 min	rt	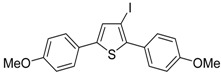	92
3	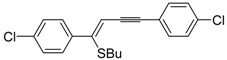	CH_2_Cl_2_	15 min	rt	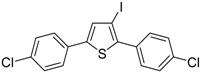	77
4	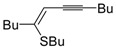	CH_2_Cl_2_	20 min	rt	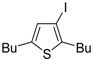	65
5	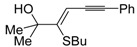	CH_2_Cl_2_	30 min	rt	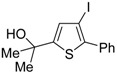	84
6	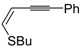	DCE	2 h	70 °C	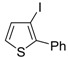	80
7	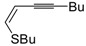	DCE	2 h	70 °C	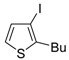	68

*^a^*: I_2_ was added dropwise in the appropriate solvent; *^b^*: Isolated yield based on starting **46**.

**Scheme 18:**
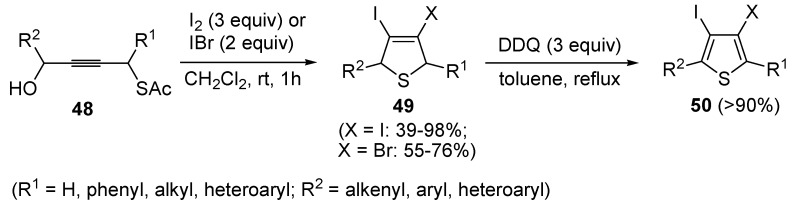
Synthesis of 3,4-dihalodihydrothiophenes **49** by iodocyclization of *S*-4-hydroxybut-2-ynyl ethanethioate **48** and their oxidation into 3,4-dihalothiophenes **50** [91,92].

3,4-Dihalodihydrothiophenes **49** have been obtained in moderate to excellent yields (39%–98%) by the iodocyclization of *S*-4-hydroxybut-2-ynyl ethanethioate **48**, carried out with an excess of I_2_ or IBr (2–3 equiv) in CH_2_Cl_2_ at rt for 1 h, according to Scheme 18 [91,92]. These products could be conveniently converted into the corresponding 3,4-dihalothiophenes **50** through oxidation with DDQ and then further elaborated by the Heck or Sonogashira reactions [91].

**Scheme 19:**
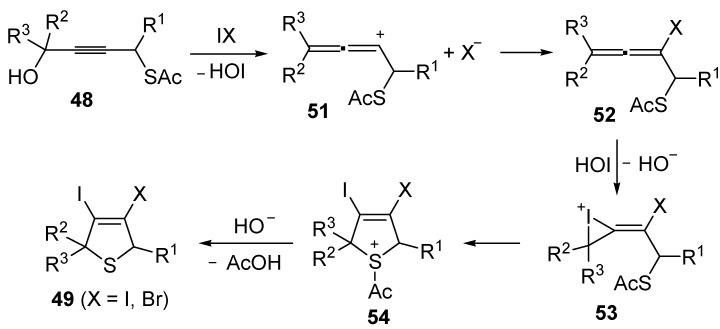
Proposed mechanism for the formation of 3,4-dihalodihydrothiophenes **49** by iodocyclization of *S*-4-hydroxybut-2-ynyl ethanethioate **48** [91,92].

Mechanistically, the idocyclization process is believed to occur through I_2_- or IBr-induced formation of an allenic carbocation **51** (with simultaneous formation of HOI), followed by iodide (or bromide) attack to give an iodoallene (or bromoallene) intermediate **52** (Scheme 19). Reaction of the latter with HOI affords the iodonium intermediate **53**, which then undergoes intramolecular nucleophilic attack by the sulfur atom to give sulfonium cation **54**. Deacylation of the latter by the previously generated hydroxide anion eventually affords 3,4-dihalodihydrothiophenes **49** (Scheme 19) [91,92].

**Scheme 20:**
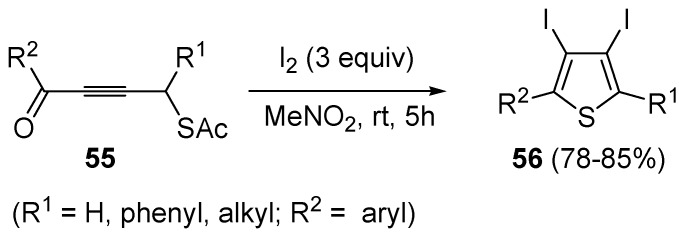
Synthesis of 3,4-diiodothiophenes **56** by iodocyclization of *S*-4-oxobut-2-ynylethanethioates **55** [92].

Starting from *S*-4-oxobut-2-ynylethanethioates **55**, the formation of 3,4-diodothiophenes **56** could be obtained directly, using 3 equiv of I_2_ in nitromethane at rt for 5 h (Scheme 20) [92].

## 4. Synthesis of Thiophene Derivatives by Carbocyclization of *S*-Containing Alkyne Substrates

**Scheme 21:**
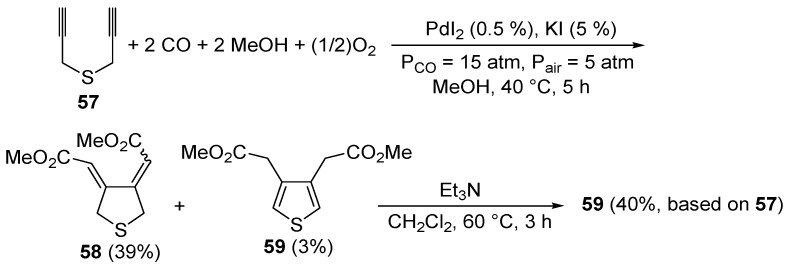
Synthesis of 3,4-*bis*(methoxycarbonylmethyl)thiophene (**59**) by PdI_2_-catalyzed oxidative carbonylative carbocyclization of dipropargyl sulfide **57** followed by base-promoted isomerization [93].

Only a few methods have been reported so far in the literature for the synthesis of thiophenes through carbocyclization of *S*-containing acetylenes. To the best of our knowledge, the first catalytic example of such an approach was reported by our research group in 1999 [93]. It involved the PdI_2_/KI-catalyzed carbonylative carbocyclization of dipropargyl sulfide (**57**) to afford a mixture of 3,4-*bis*(methoxycarbonylmethylene)tetrahydrothiophene (**58**, 39%, *Z*,*Z*:*E*,*E* *ca.* 1:1) and 3,4-*bis-* (methoxycarbonylmethyl)thiophene (**59**, 3%), which could be treated directly, without further purification, with Et_3_N in CH_2_Cl_2_ at 60 °C for 3 h to selectively give the novel thiophene derivatative **59** in 40% isolated yield based on starting **57** (Scheme 21). The carbonylation reaction was carried out in MeOH as the solvent at 40 °C and under 20 atm of a 3:1 mixture of CO-air, in the presence of 0.5 mol % of PdI_2_ and 5 mol % of KI for 5 h [93].

**Scheme 22:**
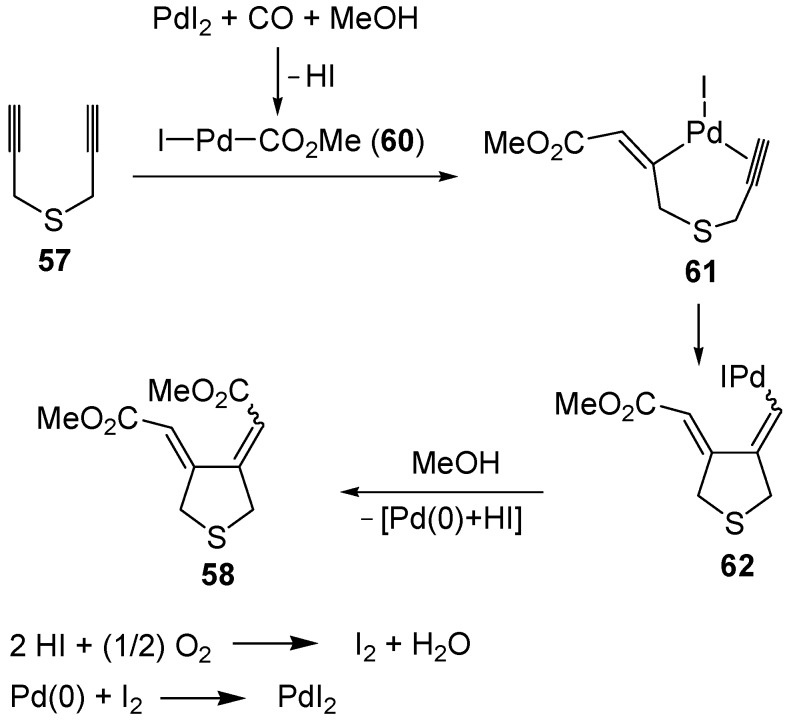
Proposed mechanism for the formation of *bis*(methoxycarbonylmethylene)-tetrahydrothiophene (**58**) by PdI_2_-catalyzed oxidative carbonylative carbocyclization of dipropargyl sulfide (**57**) [93].

The carbonylative carbocyclization process started with the formation of a methoxycarbonylpalladium iodide intermediate **60** from the reaction between PdI_2_, CO, and MeOH [49,94,95,96,97,98,99,100], followed by the insertion of the triple bond of **57** into the palladium-carbon bond to give complex **61**, stabilized by the chelation from the second triple bond (Scheme 22; anionic iodide ligands are omitted for clarity). Further insertion of the triple bond leads to the carbocyclized vinylpalladium intermediate **62**, from which the final product **58** is obtained from nucleophilic displacement by MeOH. In the last step, Pd(0) was generated, which is then reoxidized back to PdI_2_ according to a mechanism involving initial oxidation of 2 mol of HI (also ensuing from the carbonylation process) to give I_2_, followed by oxidative addition of I_2_ to Pd(0) [49,94,95,96,97,98,99,100] (Scheme 22).

**Scheme 23:**
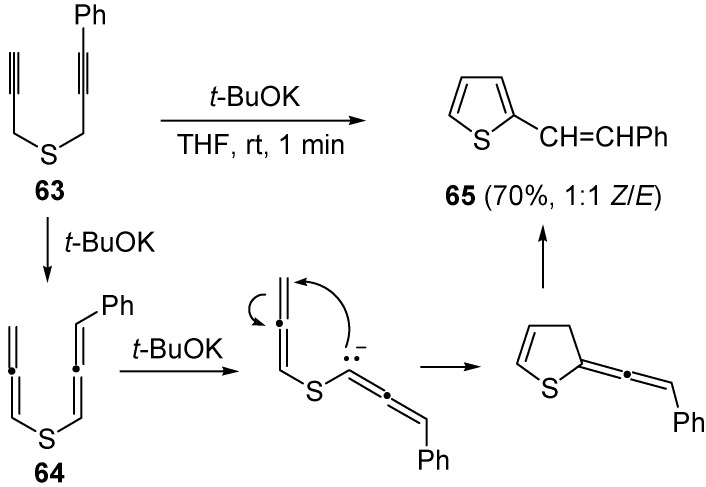
Formation of 2-styrylthiophene (**65**) by base-promoted carbocyclization of (3-phenylprop-2-ynyl)(prop-2-ynyl)sulfane (**65**) [101].

Later on, the anionic carbocyclization of some dipropargylic sulfides was studied, using *t*-BuOK in THF at rt for 1 min [101]. The reaction of (3-phenylprop-2-ynyl)(prop-2-ynyl)sulfane (**63**) led to a 1:1 diastereoisomeric mixture of 2-styrylthiophene (**65**) in 70% yield, through a mechanism involving the formation of diallenyl sulfide **64** as intermediate (Scheme 23). Similar results were obtained with (4,4-dimethylpent-2-ynyl)(3-phenylprop-2-ynyl)sulfane [101].

**Scheme 24:**
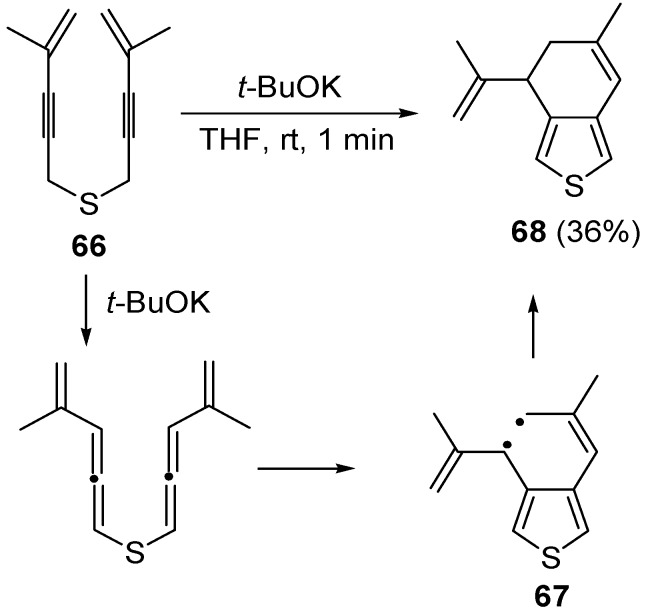
Formation of 6-methyl-4-(prop-1-en-2-yl)-4,5-dihydrobenzo[*c*]thiophene (**68**) by base-promoted carbocyclization of *bis*(4-methylpent-4-en-2-ynyl)sulfane (**66**) [101].

A more complicated reaction mixture was observed from the reaction of *bis*(3-phenylprop-2-ynyl)sulfane, with formation of products deriving from radical cycloaromatization besides the expected vinylthiophene. A radical cycloaromatization mechanism was also at work in the case of *bis*(4-methylpent-4-en-2-ynyl)sulfane (**66**) with formation of 6-methyl-4-(prop-1-en-2-yl)-4,5-dihydrobenzo[*c*]thiophene **68** in 36% yield, through the formation of the diradical intermediate **67** [101] (Scheme 24).

**Scheme 25:**
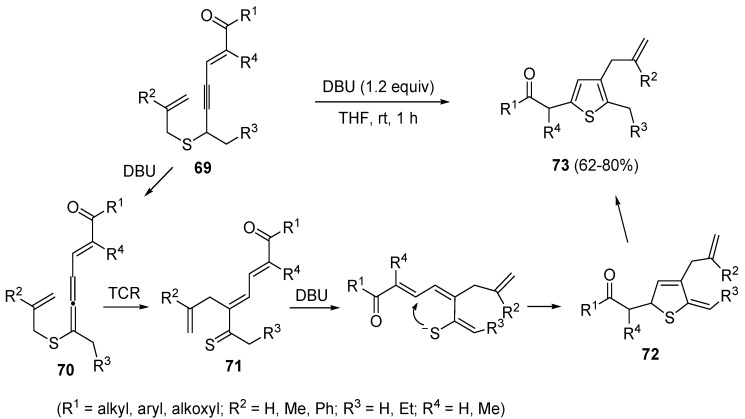
Synthesis of β-allyl thiophene derivatives **73** by base-promoted isomerization/thio-Claisen rearrangement/conjugate addition/aromatization of allyl(4-en-2-ynyl)sulfanes **69** [102].

A propargyl-allenyl isomerization was also the first step in the formation of β-allyl thiophene derivatives **73** starting from functionalized allyl(4-en-2-ynyl)sulfanes **69**, using DBU as the base in THF at rt [102]. As shown in Scheme 25, the initially formed eneallynyl intermediate **70** underwent thio-Claisen rearrangement (TCR) to give trienethione **71**. Deprotonation of the latter followed by intramolecular conjugate addition afforded 5-methylene-2,5-dihydrothiophene **72**, whose aromatization led to the final thiophene derivative **73** [102].

An interesting tandem thermal rearrangement/carbocyclization process of dipropargylic disulfides **74**, leading to a mixture of 1,3-dihydrothieno[3,4,-*c*]thiophenes **75** and thienyl disulfides **76**, has been reported recently [103]. As shown in Table 7, the reaction takes place in CHCl_3_, MeCN, or DMSO as the solvent at 60–70 °C for 1.5–160 h [103].

**Scheme 26:**
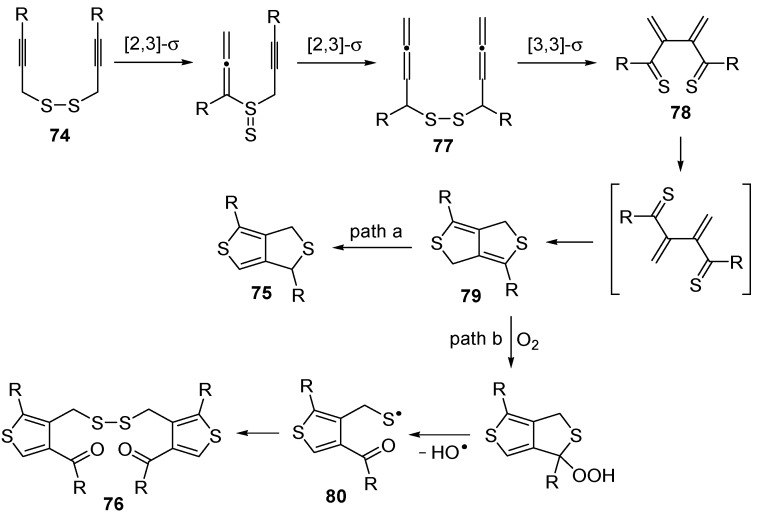
Proposed mechanistic pathway for the formation of 1,3-dihydrothieno[3,4-c]-thiophenes **75** and thienyl disulfides **76**, by tandem thermal rearrangement/carbocyclization of dipropargylic disulfides **74** [103].

Mechanistically, the reaction leading to **75** is believed to occur via an initial double [2,3]-sigmatropic rearrangement to give diallenyl disulfides **77**, which may then undergo a [3,3]-sigmatropic rearrangement to give 2,3-dimethylene-1,4-dithione **78**, followed by a double conjugate addition of the sulfur atoms to the double bonds of **78**, to give 1,4-dihydrothieno[3,4-*c*]thiophene **79**, and isomerization (Scheme 26, path a). On the other hand, **76** may be formed by dimerization of the thiyl radical intermediate **80**, formed in its turn from **79** by the action of O_2_ (Scheme 26, path b). Accordingly, the formation of **76** could be minimized working in the absence of air under argon atmosphere (entry 2, Table 8) [103].

**Table 7:** Synthesis of 1,3-dihydrothieno[3,4-*c*]thiophenes **75** and thienyl disulfides **76** by tandem thermal rearrangement/carbocyclization process of dipropargylic disulfides **74** *^a^* [103]. 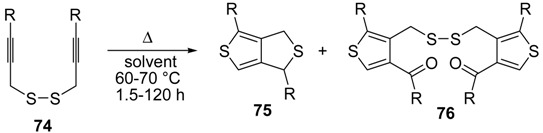

Entry	74	Solvent	t (h)	T (°C)	75	76	Yield of 75 (%) *^b^*	Yield of 76 (%) *^b^*
1	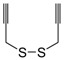	CHCl_3_	1.5	60	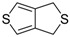	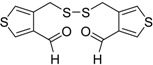	13	27
2*^c^*	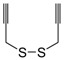	CHCl_3_	2	60	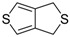	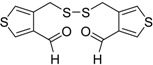	73	7
3	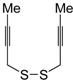	CHCl_3_	160	60	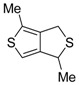	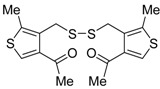	54	11
4	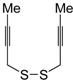	DMSO	24	70	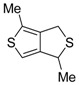	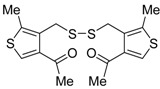	0	45
5	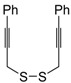	CHCl_3_	24	60	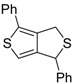	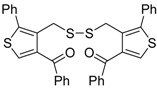	45	8
6	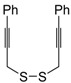	MeCN	16	60	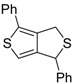	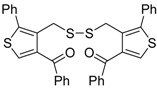	36	12

*^a^*: The mixture **75**+**76** was isolated by column chromatography. Products **75** and **76** were not separated; *^b^*: Yield based on starting **74**, referred to the isolated overall yield of **75**+**76 **and based on the **75**:**76** molar ratio as determined by ^1^H-NMR; *^c^*: The reaction was carried out under argon atmosphere.

**Table 8:** Multicomponent synthesis of ethyl 2-(2-(dimethylamino)thiophen-3-yl)-2-oxoacetate derivatives **84** starting from acetylenic esters **81**, tetramethylthiourea **82**, and ethyl bromopyruvate **83** *^a^* [104]. 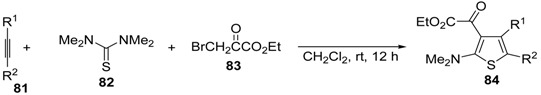

Entry	81	84	Yield of 84 *^b^* (%)
1	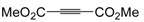	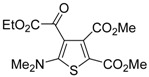	90
2	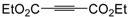	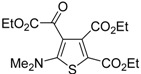	85
3	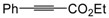	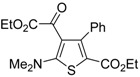	74
4	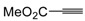	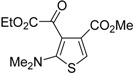	73
5	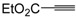	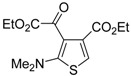	75

*^a^*: All reactions were carried out with an equimolar amount of **81**, **82**, and **83** (2 mmol per 15 mL of CH_2_Cl_2_); *^b^*: Isolated yield.

## 5. Synthesis of Thiophene Derivatives by Miscellaneous Methods Starting from Functionalized Alkyne Substrates

Miscellaneous methods that cannot be classified into the previous categories are reviewed here. Acetylenic esters can be useful precursors for the construction of the thiophene ring. Thus, ethyl 2-(2-(dimethylamino)thiophen-3-yl)-2-oxoacetate derivatives **84** have been conveniently synthesized through a multicomponent approach, employing acetylenic esters **81**, tetramethylthiourea **82**, and ethyl bromopyruvate **83** as starting materials [104]. Reactions were carried out in CH_2_Cl_2_ at rt using equimolar amounts of **81**, **82**, and **83**, to afford the corresponding thiophenes **84** in good to high yields (Table 8) [104].

**Scheme 27:**
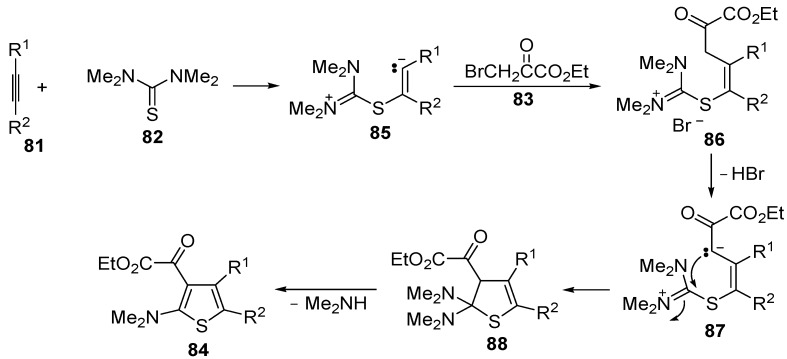
Proposed mechanism for the formation of ethyl 2-(2-(dimethylamino)thiophen-3-yl)-2-oxoacetate derivatives **84** starting from acetylenic esters **81**, tetramethylthiourea **82**, and ethyl bromopyruvate **83** [104].

The proposed reaction mechanism involves the formation of a 1,5-dipolar intermediate **85** from the reaction between **81** and **82**, followed by nucleophilic attack by the carbanion to **83** to give the organic salt **86**. The final thiophene derivative **84** is then formed from **86** by elimination of HBr to give the dipolar intermediate **87**, followed by intramolecular nucleophilic attack, affording dihydrothiophene **88**, and elimination of dimethylamine (Scheme 27) [104].

**Scheme 28:**
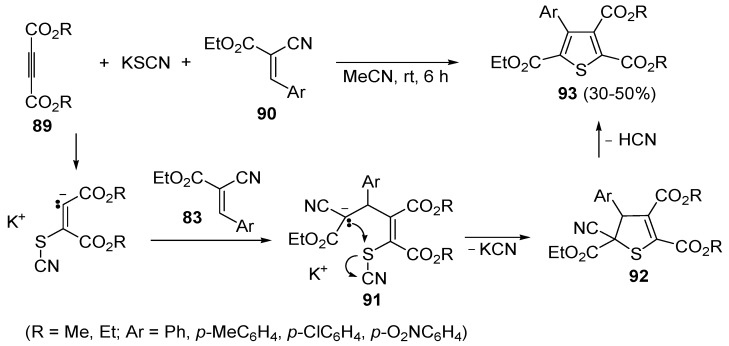
Synthesis of trialkyl 4-arylthiophene-2,3,5-tricarboxylates **93** starting from dialkyl acetylenedicarboxylates **89**, KSCN, and 3-aryl-2-cyanoacrylates **90** [105].

In a similar way, trialkyl 4-arylthiophene-2,3,5-tricarboxylates **93** were obtained in moderate yields (30%–50%) from the reaction between dialkyl acetylenedicarboxylates **89**, KSCN, and 3-aryl-2-cyanoacrylates **90**, carried out in MeCN at rt for 6 h, through the intermediate formation of the organic salt **91**, which undergoes cyclization with loss of KCN (to give dihydrothiophene derivative **92**) followed by elimination of HCN (Scheme 28) [105].

**Scheme 29:**
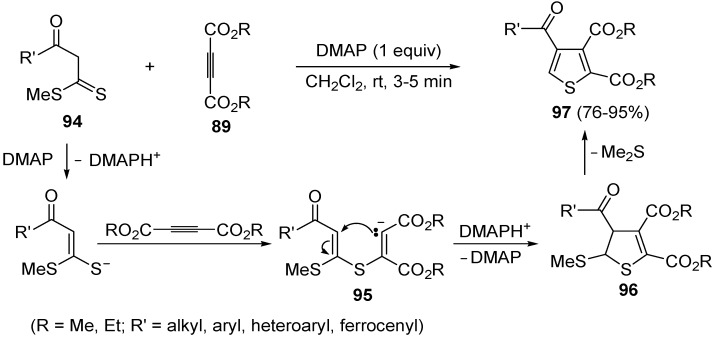
Synthesis of dialkyl thiophene-2,3-dicarboxylate derivatives **97** starting from β-oxodithioesters **94** and dialkyl acetylenedicarboxylates **89** in the presence of DMAP [106].

Another useful utilization of acetylenic diesters for the thiophene synthesis has been reported recently. It involves the reaction between β-oxodithioesters **94** and dialkyl acetylenedicarboxylates **89** (1:1 molar ratio) carried out in the presence of an equimolar amount of dimethylaminopyridine (DMAP) in CH_2_Cl_2_ at rt for 3–5 min (Scheme 29) [106]. The process takes place through α-deprotonation of **94** by DMAP and intermolecular conjugate addition (from nucleophilic attack by the sulfur atom to the triple bond of **89**, to give anionic intermediate **95**), followed by intramolecular conjugate addition to give dihydrothiophene **96**, and elimination of Me_2_S, eventually leading to dialkyl thiophene-2,3-dicarboxylate derivatives **97** (Scheme 29) [106].

**Scheme 30:**
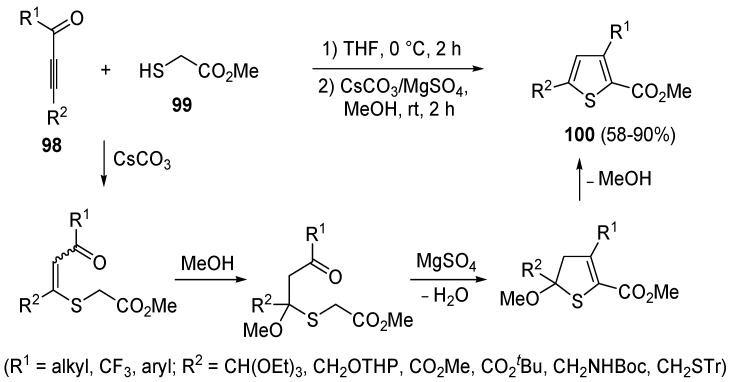
Synthesis of methyl thiophene-2-carboxylates **100** from acetylenic ketones **98** and methyl thioglycolate (**99**) in the presence of CsCO_3_, MgSO_4_, and MeOH [108].

A mechanism involving a conjugate addition by a thiolate anion to an activate triple bond was also at work in a modification of the classical Fiesselmann thiophene synthesis [107], involving the reaction between acetylenic ketones **98** and methyl thioglycolate **99** to give methyl thiophene-2-carboxylates **100** (Scheme 30) [108]. Reactions were carried out by dissolving an equimolar amount of **98** and **99** in THF at 0 °C followed, after 2 h, by the addition of a 1:2 mixture of CsCO_3_/MgSO_4_ in MeOH and stirring at rt for 2 h [108].

A particularly convenient approach to 2,4-disubstituted thiophenes **104**, based on a sequential three-component Sonogashira coupling/Fiesselmann-type cyclocondensation, has been reported recently [109]. Thus, the reaction between (hetero)aroyl chlorides **101** and terminal alkynes **102** (1.1 equiv) (carried out at rt in THF for 2 h, in the presence of 2 mol % of PdCl_2_(PPh_3_)_2_, 4 mol % of CuI, and 1.05 equiv of Et_3_N) was followed by the addition of EtOH, ethyl thioglycolate **103** (1.2 equiv) and DBU (1.5 equiv), to give, after stirring for 12–24 h at rt, thiophene derivatives **104** in moderate to excellent yields (32%–97%, Table 9). The method has also been successfully applied to the synthesis of luminescent terthiophenes and pentathiophenes [109].

**Table 9:** Synthesis of ethyl thiophene-2-carboxylates **104** starting from sequential Sonogashira coupling between (hetero)aroyl chlorides **101** and terminal alkynes **102**/Fiesselmann-type cyclocondensation *^a^* [109]. 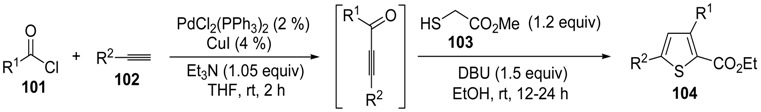

Entry	101	102	104	Yield of 104 *^b^* (%)
1	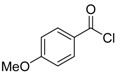	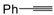	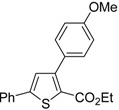	97
2	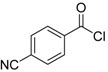	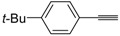	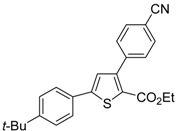	88
3	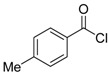	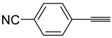	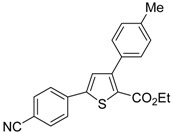	68
4	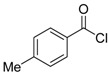	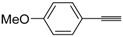	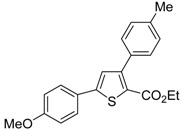	83
5	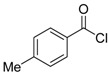	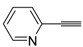	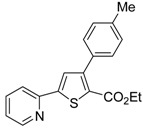	32
6	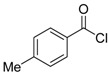	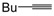	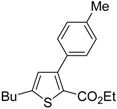	50
7	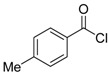	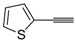	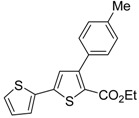	80
8	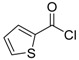	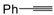	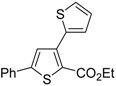	90

*^a^*: The Sonogashira coupling was carried out in THF at rt for 2 h, employing 1 equiv of **101** (0.1 M), 1.1 equiv of **102**, 0.02 equiv of PdCl_2_(PPh_3_)_2_, 0.04 equiv of CuI, and 1.05 equiv of Et_3_N. After stirring at rt for 2 h, EtOH (1 mL) was added, together with 1.2 equiv of **103** and 1.5 equiv of DBU; the resulting mixture was then allowed to stir for 12–24 h; *^b^*: Isolated yield based on starting **101**.

**Scheme 31:**
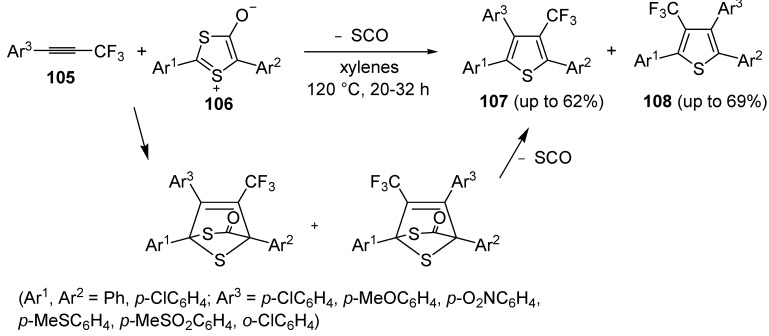
Synthesis of 2,3,5-triaryl-4-trifluoromethylthiophenes **107** and **108** from 1-aryl-3,3,3-trifluoro-1-propynes **105** and 1,3-dithiolium-4-olates **106** [110].

An interesting approach to regioisomeric 2,3,5-triaryl-4-trifluoromethylthiphenes **107** and **108**, based on 1,3-dipolar cycloaddition between 1-aryl-3,3,3-trifluoro-1-propynes **105** and 1,3-dithiolium-4-olates **106** (1:1 molar ratio), was developed some years ago (Scheme 31) [109]. Reactions were carried out in xylenes at 120 °C for 20–32 h [110].

## 6. Conclusions

The development of novel, efficient and selective methods for the construction of the thiophene ring starting from acyclic precursors is a very important target in current organic synthesis, in view of the high significance of the products obtained and of the more and more stringent requirements in the direction of a sustainable chemistry. In this regard, the synthesis of thiophene derivatives by heterocyclization of readily available *S*-containing alkyne substrates has proved to be a valuable and reliable approach, and it is destined to assume a central role in the next future for the one-step production of this particularly important class of heterocyclic derivatives. Recent progress in organometallic catalysis has also recently opened the way to the use of metal catalysis for promoting *S*-heterocyclization reactions leading to thiophenes under particularly mild and efficient reaction conditions. Further progress will allow developing other novel synthetic routes characterized by even more efficiency and selectivity under environmentally friendly conditions.
